# Increased Effect of Foot-and-Mouth Disease Virus Vaccine Structural Protein Antibody Positivity Rates in Piglets Orally Treated with Amino–Zinc Complex

**DOI:** 10.3390/ani13122027

**Published:** 2023-06-18

**Authors:** Byoung-Ryol Lee, Hu-Jang Lee, Nam-Hoon Kim, Yong-Sik Kim, Kwang Il Park

**Affiliations:** 1 Institute of Tissue Regeneration, College of Medicine, Soonchunhyang University, Cheonan 31151, Republic of Korea; ceo@bt-n.co.kr; 2 College of Veterinary Medicine, Gyeongsang National University, Jinju 52828, Republic of Korea; hujang@gnu.ac.kr; 3 ZinexBio Corporation, Asan 31538, Republic of Korea; znr02@znexbio.co.kr

**Keywords:** oral adjuvant (OAdj), piglet clinical trial, antibody positivity rate, FMD vaccine antibody titer, immunological index

## Abstract

**Simple Summary:**

Inactivated foot-and-mouth disease (FMD) vaccines are used to protect livestock against the FMD virus. However, adequate levels of antibodies to provide protection against the FMD virus cannot be rapidly produced and take a long time to develop. In this study, to solve the challenge presented by the low antibody formation following FMD vaccination in pigs, an ionic amino–zinc complex (Amino–Zn), which has high bioavailability and immunity-enhancing effects, was administered orally. The effect on the FMD vaccine structural protein (SP) antibody formation was evaluated. As a result, the FMD vaccine SP antibody titer and immune indicators (IFN-γ, IgA) were significantly higher in the FMD vaccine group administered with 0.2% Amino–Zn in the feed compared with the positive control group (FMD vaccine only) after the first and second vaccinations. These results demonstrated that administering Amino–Zn effectively improved the antibody titer induced by the FMD vaccine and the immunity of the piglets.

**Abstract:**

Foot-and-mouth disease (FMD) is a highly contagious animal disease that occurs in cloven-hoofed animals including pigs. To prevent FMD, vaccines and adjuvants are routinely used to induce an immune response; however, it requires an extended period of time to produce sufficient antibodies to prevent viral infection. In this study, we evaluated the increased effectiveness of the FMD vaccine structural protein (SP) antibody by administrating the Amino–Zn adjuvant to 100 pigs from 3 test pig farms in their feed. The FMD vaccine antibody titer and immunological index were analyzed using an enzyme-linked immunosorbent assay (ELISA) kit, and the hematological and blood biochemical parameters were analyzed using an automatic blood analyzer. The titer of the FMD vaccine SP antibodies in the 0.2% Amino–Zn-administered group was significantly increased compared to that of the positive control group only injected with FMD vaccine at 4 weeks after the first vaccination and at 4, 8, and 16 weeks after the second vaccination (*p* < 0.05). The FMD vaccine SP antibody positive rate was 100% until shipment. The IFN-γ and IgA levels were significantly increased by Amino–Zn administration 4 weeks after the first vaccination and 4 weeks after the second vaccination (*p* < 0.05). On the other hand, serum AST, and CPK (*p* < 0.001) were significantly decreased by Amino–Zn administration. These results show that the administration of Amino–Zn is effective in enhancing the antibody titer and immunogenicity of the FMD vaccine and can be used as an oral adjuvant (OrAd) to prevent viral diseases, such as FMD.

## 1. Introduction

Foot-and-mouth disease (FMD) is a highly contagious disease that occurs in animals with split hooves, such as cattle, pigs, and goats [1]. Foot-and-mouth disease virus (FMDV) belongs to the family and genus *Picornaviridae* and *Aphthovirus,* respectively, and is a single-stranded positive RNA virus [2]. FMDV has high antigenic variability, currently consisting of 80 subtypes and 7 major serotypes, which are A, O, C, Asia1, SAT1, SAT2, and SAT3 [3].

The highly contagious FMDV spreads rapidly through direct contact with infected animals and indirect contact through the air and humans [1,4]. Livestock that are infected with FMDV experience fever and exhibit symptoms, such as the formation of blisters on the gums, tongue, and nose, loss of appetite, as well as limping [1,5]. The transmission power, and growth, movement, and lactation disorders caused by FMDV lead to a decrease in productivity and cause enormous economic damage to livestock farmers and the country [6,7].

To prevent FMDV, most countries use the inactivated whole virus FMD vaccine with an immune adjuvant [8]. To prevent FMD, the Korean government is implementing a prevention policy by vaccinating against FMDV twice at an interval of one month. However, even with the administration of the FMD vaccine and included adjuvant, an effective immune response takes a long time to develop sufficient antibodies to be effective [9]. With the administration of immune adjuvants into the subcutaneous tissue, there is a challenge in restoring the status as an FMD free country due to the occurrence of inflammation, allergies, suppuration, and other adverse reactions at the injection site [8].

Zinc, which is essential for growth, development, and the maintenance of the immune system, plays a role in regulating innate and acquired immunity through the proliferation and maturation of the immune cells [9]. In weaned piglets, if they do not receive sufficient zinc through their feed, the immune system malfunctions, and subsequent pathogen-induced diarrhea can lead to death [10,11]. This is because the passive immunity is reduced in weaned piglets after lactation is halted and active immunity has not been sufficiently developed, which results in a lower resistance to pathogen infection [12,13].

An amino–zinc complex (Amino–Zn) is ionic; it combines with amino acids (AAs) through atom sharing and has high bioavailability compared to other inorganic minerals [14]. Aspartic acid (ASP) is used as a precursor to synthesize essential AAs, such as lysine and methionine, and exhibits a stable structure by binding to zinc through a carboxyl or amine group [15]. The oral ingestion of Amino–Zn has been reported to improve the effect of immunity and productivity [14]. However, there are no studies regarding the vaccine antibody formation promoting effect after Amino–Zn administration, which enhances immune cell activity.

Therefore, the purpose of this study was to evaluate the effectiveness of oral Amino–Zn adjuvant administered in piglets by specifically assessing the antibody titer and blood biochemical analysis after FMD vaccination. The antibody positivity rate according to the titer and period of vaccination was evaluated to confirm the applicability of Amino–Zn as an oral adjuvant to increase the antibody titer induced by the FMD vaccine.

## 2. Materials and Methods

This study was conducted with the approval of the Korean Agriculture, Forestry, and Livestock Quarantine Headquarters for veterinary medicine clinical trials. All animal experimental processes were approved by the Institutional Animal Care and Use Committee (IACUC) of Gyeongsang National University, approval number GNU-211013-P0084, and performed in accordance with the guidelines of the IACUC of Gyeongsang National University, Republic of Korea.

### 2.1. Test Farm, Group, and Animals

A general breeding farm in Hapcheon, Gyeongsangnam-do, was selected as a test farm, and 100 healthy piglets (Duroc × Yorkshire × Landrace) before and after 6 weeks of age were selected and identified by applying a mark. The test animals were included in the experiment after acclimatization for one week in the experimental breeding facility. The test animals were randomly assigned to one of the following five treatments (n = 20 per group): NC (negative control; unvaccinated and no additional added Amino–Zn to diet), PC (positive control; vaccination only), ZA-0.1 (vaccination + 0.1% Amino–Zn dietary intake), ZA-0.2 (vaccination + 0.2% Amino–Zn dietary intake), and ZA-0.3 (vaccination + 0.3% Amino–Zn dietary intake). Amino–Zn was administered in a mixture of 0.1, 0.2, or 0.3% in the feed according to the test group, for 10 weeks from 2 weeks before the first FMD vaccine to 4 weeks after the second FMD vaccine. The feed was made to meet the nutrient recommendations of the National Research Council (NRC, 2012). Each treatment group was allowed to feed ad libitum through an automatic feeder and water was freely available. The relative humidity in the pig house was controlled at 50–60% and the temperature was gradually reduced from 28 to 22 °C.

### 2.2. Vaccination

The FMD vaccine used in this experiment was the inactivated purified bivalent FMD vaccine Bioatogen [Careside, Korea]. According to the composition of the test group, the piglets were inoculated with 2 mL intramuscular injection at 8 and 12 weeks of age.

### 2.3. Measurement of Structural Protein (SP) Antibody Titer

To measure the antibody titer following FMD vaccination, the blood from the experimental pigs of each farm was collected before the first vaccination (8 weeks old), before the second vaccination (12 weeks old), 4 weeks after the second vaccination (16 weeks old), 8 weeks after the second vaccination (20 weeks of age), and at the time of shipment (28 weeks of age).

To measure the FMD vaccine-induced antibody titer, the serum was separated, and an LPB ELISA kit (Biogenesis-Bago SA, Garin, Argentina) for the Bioatogen FMD vaccine O type was used. The antibody titers were determined by measuring the absorbance at wavelengths of 405/415 nm in accordance with the manufacturer’s instructions. To determine the antibody titer, a standard curve was generated by measuring the absorbance of the standard serum (control) at various dilution factors. Subsequently, the antibody titer was calculated by comparing the absorbance value of the serum sample to the standard curve.

### 2.4. Cellular Immune Marker (IFN-γ) Test

Serum IFN-γ concentration was measured using the Nori Porcine IFN-γ ELISA kit (Genorise Scientific Inc., Glen Mills, PA, USA), and analyzed in accordance with the manufacturer’s instructions.

### 2.5. Serum Immunoglobulin (IgG, IgM, and IgA) Measurement

The concentrations of the IgG, IgM, and IgA in the serum were measured using the Porcine IgG ELISA kit (Koma Biotech, Seoul, Republic of Korea), Porcine IgM ELISA kit (Koma Biotech), and Porcine IgA ELISA kit (Koma Biotech), respectively. Analysis was performed according to the manufacturer’s protocol.

### 2.6. Hematological and Blood Biochemical Analysis

Blood collected from the experimental pigs was analyzed for hematological and biochemical indicators using an automatic blood analyzer (Hitachi 911 chemistry analyzer, Hitachi, Japan). Hematological analyses included the measurement of white blood cells (WBCs), hemoglobin (Hb), hematocrit (HCT), lymphocytes (LYMs), neutrophils (NEUs), eosinophils (EOSs), basophils (BASs), monocytes (MONs), mean corpuscular volume (MCV), mean corpuscular hemoglobin (MCH), mean corpuscular hemoglobin concentration (MCHC), and platelets (PLTs).

The blood biochemical analyses included the measurement of alkaline phosphatase (ALP), glucose (GLU), aspartate aminotransferase (AST), alanine aminotransferase (ALT), blood urea nitrogen (BUN), creatinine (CREA), and creatine phosphokinase (CPK).

### 2.7. Statistical Analysis

Data are presented as mean ± standard deviation. The average values were analyzed through one-way analysis of variance using SPSS statistics 26 software. The Duncan method was used for multiple comparisons of the differences between groups. Difference were considered significant when *p* < 0.05.

## 3. Results

### 3.1. Effect of Amino–Zn on the FMD Vaccine Antibody Titer

The blood of the experimental pigs was collected before vaccination (8 weeks of age), 4 weeks after the first vaccination (12 weeks of age), 4 weeks of the second vaccination (16 weeks of age), 8 weeks (20 weeks of age), and before shipment (28 weeks of age). The antibody positivity rate was evaluated after measuring the antibody titer induced by the FMD vaccine using the LPB ELISA kit (Biogenesis-Bago SA) for the Bioatogen FMD vaccine type O (Table 1 and Table 2). Co-administration of the FMD vaccine and Amino–Zn increased the vaccine antibody titer, and when Amino–Zn was mixed with feed at concentrations of 0.2% and 0.3%, the increase was statistically significant compared to the positive control group (*p* < 0.05). The antibody positivity rate in the Amino–Zn combination group was higher than that of the positive control group vaccinated 4 weeks after the primary vaccination, and when 0.2% Amino–Zn was administered, the antibody positivity rate was 90%, which was 45% higher than that of the positive control group (95% confidence interval, 57.6–89.9). After the second FMD vaccination, all groups except the negative control (unvaccinated) group showed an antibody positivity rate of 100%.

### 3.2. Effect of Amino–Zn on Cellular Immune Markers (IFN-γ)

To confirm the effect of the combined administration of the FMD vaccine and Amino–Zn on the cellular immune markers, IFN-γ was analyzed using an ELISA kit (Genorise Scientific Inc., Glen Mills, PA, USA) (Table 3).

The combination of FMD vaccine and Amino–Zn was confirmed to increase the IFN-γ concentration in the blood. In particular, 4 weeks after the first and second dose of FMD vaccine, the TRT3 (ZA-0.3) and TRT2 (ZA-0.2) groups had the highest blood IFN-γ concentrations. In addition, the TRT2 (ZA-0.2) and TRT3 (ZA-0.3) groups significantly upregulated the IFN-γ compared to the positive control group (*p* < 0.05 and *p* < 0.01, respectively).

### 3.3. Effect of Amino–Zn on the Humoral Immune Markers (IgG, IgM, and IgA)

To confirm the efficacy of the combined administration of the FMD vaccine and Amino–Zn on the humoral immune markers, the IgG, IgM, and IgA were each evaluated using the Porcine IgG ELISA kit (Koma Biotech), Porcine IgM ELISA kit (Koma Biotech), and Porcine IgA ELISA kit (Koma Biotech), respectively (Table 4.). The concentrations of IgG, IgM, and IgA in the blood increased with FMD vaccination, and showed a tendency to decrease in the negative control group where no vaccine or Amino–Zn was administered. The combined treatment of the FMD vaccine and Amino–Zn reduced the concentration of IgG and IgM in the blood but did not demonstrate a significant difference. On the other hand, the concentration of IgA was significantly increased in the TRT2 (ZA-0.2) (*p* < 0.05) and TRT3 (ZA-0.3) (*p* < 0.01) groups treated with the FMD vaccine and Amino–Zn.

### 3.4. Effect of Amino–Zn on Hematological Indicators

Blood was collected from the experimental pigs and the hematological indicators were analyzed using an automatic blood analyzer (Table 5). Regarding the hematological parameters, the FMD vaccine and Amino–Zn combination did not show statistically significant differences when compared with the positive control group.

### 3.5. Effect of Amino–Zn on Blood Biochemical Markers

Blood biochemical parameters were analyzed using an automatic blood analyzer on the serum obtained from the experimental pigs before FMD vaccination, 4 weeks after the first vaccination, and 4 weeks after the second vaccination (Table 6).

The combined treatment of FMD vaccine and Amino–Zn significantly reduced the concentrations of AST and CPK compared to the positive control group, but there was no statistically significant differences in the other blood biochemical parameters.

## 4. Discussion

The vaccine used for FMD prevention was administered by inoculation with the inactivated FMDV antigen [8]. However, when pigs are vaccinated with inactivated FMD antigen vaccine, although FMD antibodies are generated within 1–2 weeks, they fail to maintain long-term immunity, necessitating an additional vaccine administration and the use of immune adjuvants [16]. Zinc, which is used as a vaccine immune adjuvant, is utilized in the swine industry for the purposes of disease prevention and productivity enhancement. Amino–Zn, composed of zinc and AAs in a chelated form, has been shown to exhibit effects, such as increased blood zinc levels and improved growth rate, when administered to pigs [14]. Furthermore, Amino–Zn has been reported to have a positive impact on the function and proliferation of T and B cells [17]. Therefore, in this study, we investigated the synergistic effect of the combined administration of the FMD vaccine and Amino–Zn to enhance the antibody titer by stimulating the immune cells and providing the necessary signals for antibody production.

To prevent FMD, it is crucial to increase antibody levels and antibody positivity rate by administering at least the primary vaccine dose, thereby reducing the risk of disease occurrence. In this study, we administered the FMD vaccine and Amino–Zn concurrently to six-week-old piglets. The results showed that the first vaccine dose alone achieved a 95% antibody positivity rate. In contrast, the antibody positivity rate of the group that received only the vaccine was 75%, lower compared to the Amino–Zn administration group. These findings indicate that the combined administration of FMD vaccine and Amino–Zn enhanced the immune response triggered by the vaccine, promoted antibody production, and potentially reduced the incidence of FMD from 25% to 5%.

The co-administration with Amino–Zn significantly increased the IFN-γ levels, an important cytokine involved in the activation and regulation of various immune cells, including T cells and natural killer cells, and is known to promote the antibody production in B cells [18]. Therefore, the induction of IFN-γ by Amino–Zn administration is considered to stimulate B cells and facilitate the production of antibodies against the inactivated FMDV antigens that have been introduced into the body, thus, inducing a significant increase in FMD vaccine antibodies [19].

When the pigs were vaccinated against FMD with the co-administration of Amino–Zn, it was confirmed that the serum IgA concentration was significantly higher than that of the positive control (vaccination only) group. This result suggests that Amino–Zn administration can have a positive effect on the immune response induced by the FMD vaccine [20,21]. In addition, the increased serum IgA levels observed in the Amino–Zn administration group may be attributed to the essential role of zinc in the maturation and activation of B lymphocytes involved in IgA production. These results suggest that the administration of Amino–Zn may provide potential benefits by enhancing the immune response to vaccines and improving antibody production. However, further studies are required to explore the underlying mechanism by which Amino–Zn influences the immune responses and to evaluate the potential benefits of zinc in different vaccination situations.

Zinc is a crucial element that is bound to proteins, such as serum albumin or intracellular metallothionein and is present in all cells [22]. In our study, we did not observe any significant changes in the hematological indicators following Amino–Zn administration. However, we found a significant decrease in AST and CPK levels through blood biochemical analysis after Amino–Zn administration. AST and CPK are enzymes that play important roles in various tissues. AST is primarily found in hepatocytes, while CPK is present in the skeletal muscle, myocardium, and brain [23,24]. When tissues are damaged, AST and CPK are released into the bloodstream, leading to an increase in their levels. The lower AST and CPK levels observed in the Amino–Zn administration group suggests a potential protective effect on hepatocytes and muscle tissues. It is possible that Amino–Zn administration contributed to the reduced release of these enzymes into the bloodstream, indicating a mitigating effect on tissue damage caused by FMD vaccination.

In this study, we evaluated the effects of Amino–Zn in pigs vaccinated against FMD. However, additional research is required to assess and compare the effects of Amino–Zn in different vaccination scenarios. Conducting experiments with different vaccine types and administration timing would provide a clearer assessment of the potential benefits and applicability of Amino–Zn.

## 5. Conclusions

The results of this study showed that after oral administration of Amino–Zn to piglets and only one FMD vaccination dose, a 90% antibody positivity rate was observed, which showed that administration increased the antibody positivity rate by 45% compared to the non-Amino–Zn-dosed group (FMD vaccine only). This suggests that Amino–Zn can be used as an oral vaccine adjuvant, although further studies are required to elucidate the mechanism of immune activation. If the Amino–Zn oral administration provides sufficient protection against FMDV with only one vaccination event, the stress in pigs and occurrence of abnormal meat at the inoculation site caused by an additional vaccination may be eliminated to increase the economic feasibility of breeding farms.

## Figures and Tables

**Table 1:** Effect of the increased foot-and-mouth disease virus vaccine structural protein antibody positive rates in piglets through the co-administration with Amino–Zn.

Foot-and-Mouth Disease Antibody Titer (%)						
Group		NC	PC	TRT1	TRT2	TRT3
Before vaccination First vaccination at (8 weeks old)		21.75 ± 2.86	22.10 ± 3.75	21.90 ± 3.66	22.51 ± 3.86	22.04 ± 3.25
Primary vaccination	After 4 weeks (12 weeks old)	22.46 ± 3.92	51.42 ± 4.05	52.25 ± 3.60	54.36 ± 3.81 ^a^	55.16 ± 4.19 ^a^
	After 4 weeks (16 weeks old)	23.14 ± 4.29	65.27 ± 4.86	66.38 ± 3.41	68.45 ± 4.36 ^a^	69.04 ± 5.10 ^a^
Secondary vaccination at 12 weeks old	After 8 weeks (20 weeks old)	22.32 ± 3.76	70.67 ± 4.97	72.48 ± 4.40	73.82 ± 4.54 ^a^	73.92 ± 4.76 ^a^
	After 16 weeks (before shipment)	22.63 ± 3.10	66.94 ± 4.25	67.61 ± 3.72	69.12 ± 4.56 ^a^	69.49 ± 4.65 ^a^

^a^ Different superscripts within a row indicate a significant difference (*p* < 0.05). NC, negative control; PC, positive control; TRT1, ZA-0.1 (vaccination + 0.1% Amino–Zn dietary intake); TRT2, ZA-0.2 (vaccination + 0.2% Amino–Zn dietary intake); TRT3, ZA-0.3 (vaccination + 0.3% Amino–Zn dietary intake). Sample size n = 20.

**Table 2:** Antibody positivity induced by foot-and-mouth disease vaccination and with co-administration of Amino–Zn.

Foot-and-Mouth Disease Antibody Positive Rate (%)						
Group		NC	PC	TRT1	TRT2	TRT3
Before vaccination First vaccination at (8 weeks old)		0	0	0	0	0
Primary vaccination	After 4 weeks (12 weeks old)	0	55	65	90	85
	After 4 weeks (16 weeks old)	0	100	100	100	100
Secondary vaccination at 12 weeks old	After 8 weeks (20 weeks old)	0	100	100	100	100
	After 16 weeks (before shipment)	0	100	100	100	100

NC, negative control; PC, positive control; TRT1, ZA-0.1 (vaccination + 0.1% Amino–Zn dietary intake); TRT2, ZA-0.2 (vaccination + 0.2% Amino–Zn dietary intake); TRT3, ZA-0.3 (vaccination + 0.3% Amino–Zn dietary intake). Sample size n = 20.

**Table 3:** Effect of the combined administration of foot-and-mouth disease vaccine and Amino–Zn on the humoral immune indicators.

Group	IFN-γ Concentration (pg/mL)				
	NC	PC	TRT1	TRT2	TRT3
Before vaccination	85.8 ± 6.3	85.3 ± 5.6	86.2 ± 7.9	86.5 ± 7.8	87.1 ± 8.3
4 weeks after first vaccination	88.3 ± 9.0	97.21 ± 5.94	104.63 ± 6.92	105.24 ± 7.55 ^a^	108.72 ± 7.30 ^b^
4 weeks after second vaccination	91.1 ± 7.4	105.07 ± 7.14	115.41 ± 9.01	118.45 ± 9.82 ^a^	117.32 ± 8.67 ^a^

^a,b^ Different superscripts within a row indicate a significant difference (^a^: *p* < 0.05, ^b^: *p* < 0.01). NC, negative control; PC, positive control; TRT1, ZA-0.1 (vaccination + 0.1% Amino–Zn dietary intake); TRT2, ZA-0.2 (vaccination + 0.2% Amino–Zn dietary intake); TRT3, ZA-0.3 (vaccination + 0.3% Amino–Zn dietary intake). Sample size n = 20.

**Table 4:** Effect of the combined administration of foot-and-mouth disease vaccine and Amino–Zn on cellular immune indicators (IgG, IgM, and IgA).

**Group**	**IgG (mg/mL)**				
	**NC**	**PC**	**TRT1**	**TRT2**	**TRT3**
Before vaccination	21.73 ± 1.03	21.12 ± 0.81	21.55 ± 1.19	21.96 ± 1.29	21.85 ± 1.05
4 weeks after first vaccination	20.80 ± 0.72	24.54 ± 1.39	24.30 ± 1.16	24.41 ± 0.98	24.10 ± 0.79
4 weeks after second vaccination	20.21 ± 1.02	25.89 ± 1.08	25.11 ± 1.13	25.47 ± 1.06	25.28 ± 1.19
**Group**	**IgM (mg/mL)**				
	**NC**	**PC**	**TRT1**	**TRT2**	**TRT3**
Before vaccination	2.48 ± 0.14	2.50 ± 0.16	2.45 ± 0.09	2.47 ± 0.12	2.43 ± 0.13
4 weeks after first vaccination	20.80 ± 0.72	24.54 ± 1.39	24.30 ± 1.16	24.41 ± 0.98	24.10 ± 0.79
4 weeks after second vaccination	20.21 ± 1.02	25.89 ± 1.08	25.11 ± 1.13	25.47 ± 1.06	25.28 ± 1.19
**Group**	**IgA (mg/mL)**				
	**NC**	**PC**	**TRT1**	**TRT2**	**TRT3**
Before vaccination	0.73 ± 0.05	0.74 ± 0.06	0.71 ± 0.07	0.72 ± 0.04	0.75 ± 0.07
4 weeks after first vaccination	0.58 ± 0.04	0.84 ± 0.08	0.92 ± 0.10	0.98 ± 0.11 ^a^	1.03 ± 0.12 ^b^
4 weeks after second vaccination	0.47 ± 0.06	0.94 ± 0.09	1.04 ± 0.10	1.10 ± 0.14 ^a^	1.16 ± 0.12 ^b^

^a,b^ Different superscripts within a row indicate a significant difference (^a^: *p* < 0.05, ^b^: *p* < 0.01). NC, negative control; PC, positive control; TRT1, ZA-0.1 (vaccination + 0.1% Amino–Zn dietary intake); TRT2, ZA-0.2 (vaccination + 0.2% Amino–Zn dietary intake); TRT3, ZA-0.3 (vaccination + 0.3% Amino–Zn dietary intake). Sample size n = 20.

**Table 5:** Effect of the concomitant administration of foot-and-mouth disease vaccine and Amino–Zn on the hematological indicators.

Hematological Indicator	Group	Before Vaccination	4 Weeks after First Vaccination	4 Weeks after Second Vaccination
WBCs (×10^3^/μL)	NC	14.91 ± 1.16	14.84 ± 1.04	14.87 ± 1.37
	PC	14.96 ± 1.46	16.43 ± 1.57	15.55 ± 1.30
	TRT1	14.83 ± 1.09	15.96 ± 1.14	15.09 ± 1.16
	TRT2	14.89 ± 1.06	16.14 ± 1.27	15.23 ± 1.26
	TRT3	14.84 ± 1.20	16.09 ± 1.28	15.31 ± 1.05
RBCs (×10^6^/μL)	NC	6.82 ± 0.50	6.87 ± 0.51	6.84 ± 0.62
	PC	6.86 ± 0.38	6.83 ± 0.45	6.94 ± 0.47
	TRT1	6.92 ± 0.47	7.03 ± 0.48	7.15 ± 0.48
	TRT2	6.88 ± 0.37	7.08 ± 0.51	7.19 ± 0.46
	TRT3	6.80 ± 0.42	7.06 ± 0.41	7.22 ± 0.47
Hb (g/dL)	NC	12.05 ± 1.08	12.09 ± 1.13	12.04 ± 1.04
	PC	12.01 ± 0.81	11.93 ± 1.20	12.28 ± 1.32
	TRT1	12.06 ± 1.39	12.16 ± 0.89	12.34 ± 1.03
	TRT2	12.09 ± 1.25	12.22 ± 1.00	12.37 ± 1.25
	TRT3	12.02 ± 0.99	12.27 ± 0.90	12.44 ± 1.09
HCT (%)	NC	35.55 ± 2.74	35.43 ± 2.36	35.49 ± 3.03
	PC	35.35 ± 2.24	35.73 ± 2.62	35.91 ± 2.21
	TRT1	35.26 ± 2.69	35.68 ± 2.48	35.82 ± 2.41
	TRT2	35.49 ± 2.33	35.78 ± 2.77	35.98 ± 3.46
	TRT3	35.33 ± 2.44	35.71 ± 2.08	35.91 ± 2.44
LYMs (%)	NC	53.41 ± 2.09	53.53 ± 3.55	53.44 ± 2.97
	PC	53.67 ± 3.22	56.85 ± 3.33	57.15 ± 2.74
	TRT1	53.57 ± 3.37	54.75 ± 3.20	55.23 ± 4.09
	TRT2	53.36 ± 4.51	55.16 ± 3.52	55.62 ± 3.68
	TRT3	53.21 ± 4.12	55.35 ± 3.60	55.99 ± 3.16
NEUs (%)	NC	36.37 ± 1.32	36.69 ± 1.54	36.58 ± 1.96
	PC	36.64 ± 1.97	42.73 ± 2.05	40.51 ± 1.87
	TRT1	36.56 ± 1.77	41.85 ± 1.88	38.93 ± 1.71
	TRT2	36.34 ± 1.98	41.42 ± 2.24	38.56 ± 2.18
	TRT3	36.19 ± 1.03	41.18 ± 2.09	38.21 ± 2.06
EOSs (%)	NC	2.25 ± 0.24	2.30 ± 0.17	2.33 ± 0.20
	PC	2.28 ± 0.20	3.95 ± 0.23	3.82 ± 0.19
	TRT1	2.21 ± 0.22	3.84 ± 0.21	3.71 ± 0.23
	TRT2	2.27 ± 0.17	3.92 ± 0.23	3.77 ± 0.22
	TRT3	2.34 ± 0.19	3.78 ± 0.22	3.65 ± 0.18
BASs (%)	NC	0.79 ± 0.07	0.81 ± 0.06	0.78 ± 0.05
	PC	0.81 ± 0.05	1.24 ± 0.10	1.17 ± 0.09
	TRT1	0.83 ± 0.06	1.21 ± 0.12	1.09 ± 0.08
	TRT2	0.80 ± 0.06	1.19 ± 0.08	1.15 ± 0.06
	TRT3	0.82 ± 0.04	1.23 ± 0.11	1.16 ± 0.10
MONs (%)	NC	3.50 ± 0.26	3.59 ± 0.37	3.54 ± 0.21
	PC	3.48 ± 0.22	3.85 ± 0.30	3.72 ± 0.22
	TRT1	3.55 ± 0.30	3.77 ± 0.29	3.69 ± 0.32
	TRT2	3.49 ± 0.31	3.74 ± 0.33	3.71 ± 0.27
	TRT3	3.54 ± 0.32	3.82 ± 0.32	3.75 ± 0.34
MCV (fl)	NC	60.30 ± 2.79	59.84 ± 2.11	60.87 ± 3.27
	PC	60.79 ± 3.03	59.45 ± 2.86	60.35 ± 3.06
	TRT1	60.42 ± 2.10	59.60 ± 2.32	60.46 ± 2.01
	TRT2	60.63 ± 3.75	59.46 ± 2.58	60.30 ± 2.51
	TRT3	60.28 ± 2.68	59.37 ± 2.88	60.37 ± 2.93
MCH (pg)	NC	18.32 ± 0.92	18.63 ± 0.82	18.47 ± 0.77
	PC	18.48 ± 0.88	18.16 ± 1.04	18.57 ± 1.10
	TRT1	18.54 ± 0.70	18.04 ± 0.59	18.52 ± 0.74
	TRT2	18.34 ± 1.03	17.98 ± 0.79	18.47 ± 0.80
	TRT3	18.36 ± 0.71	18.17 ± 0.72	18.55 ± 0.97
MCHC (g/dL)	NC	32.12 ± 0.92	32.29 ± 0.85	32.20 ± 0.96
	PC	32.21 ± 1.09	32.57 ± 0.93	32.82 ± 0.85
	TRT1	32.32 ± 0.57	32.49 ± 0.80	32.72 ± 1.00
	TRT2	32.41 ± 1.00	32.51 ± 0.97	32.80 ± 0.80
	TRT3	32.42 ± 0.93	32.55 ± 0.95	32.79 ± 0.88
PLTs (×10^3^/μL)	NC	352.0 ± 23.9	349.2 ± 21.7	353.1 ± 19.9
	PC	351.2 ± 26.6	384.5 ± 25.6	373.2 ± 25.1
	TRT1	350.9 ± 19.7	385.6 ± 17.6	368.0 ± 27.1
	TRT2	354.2 ± 18.5	383.2 ± 14.6	370.8 ± 16.3
	TRT3	357.5 ± 16.9	388.8 ± 14.6	368.5 ± 23.7

WBCs, white blood cells; Hb, hemoglobin; HCT, hematocrit; LYMs, lymphocytes; NEUs, neutrophils; EOSs, eosinophils; BASs, basophils; MONs, monocytes; MCV, mean corpuscular volume; MCH, mean corpuscular hemoglobin; MCHC, mean corpuscular hemoglobin concentration; PLTs, platelets; NC, negative control; PC, positive control; TRT1, ZA-0.1 (vaccination + 0.1% Amino–Zn dietary intake); TRT2, ZA-0.2 (vaccination + 0.2% Amino–Zn dietary intake); TRT3, ZA-0.3 (vaccination + 0.3% Amino–Zn dietary intake). Sample size n = 20.

**Table 6:** Effect of concomitant administration of foot-and-mouth disease vaccine and Amino–Zn on the blood biochemical indicators.

Biochemical Indicators	Group	Before Vaccination	4 Weeks after First Vaccination	4 Weeks after Second Vaccination
ALP (U/L)	NC	237.0 ± 12.3	236.7 ± 9.35	238.2 ± 12.2
	PC	238.4 ± 13.5	247.5 ± 12.7	243.9 ± 15.8
	TRT1	235.3 ± 13.2	246.9 ± 13.1	241.2 ± 12.7
	TRT2	237.5 ± 11.5	247.1 ± 12.0	241.7 ± 13.4
	TRT3	239.8 ± 13.7	249.7 ± 15.9	242.1 ± 11.9
GLU (mg/dL)	NC	124.4 ± 11.6	125.3 ± 8.0	123.6 ± 10.4
	PC	123.9 ± 8.1	129.5 ± 7.9	127.1 ± 15.4
	TRT1	124.8 ± 12.2	127.3 ± 8.4	126.0 ± 9.4
	TRT2	123.2 ± 11.3	126.6 ± 8.7	124.3 ± 7.1
	TRT3	124.1 ± 17.7	125.1 ± 8.4	124.1 ± 10.1
AST (U/L)	NC	60.06 ± 2.82	60.20 ± 2.09	59.92 ± 3.20
	PC	60.25 ± 2.38	72.14 ± 3.45	68.43 ± 3.03
	TRT1	59.96 ± 3.09	67.03 ± 2.76 ^a^	63.14 ± 2.69 ^b^
	TRT2	60.19 ± 2.07	65.47 ± 3.73 ^b^	62.92 ± 3.90 ^a^
	TRT3	60.10 ± 2.93	64.36 ± 2.72 ^c^	62.60 ± 2.75 ^b^
ALT (U/L)	NC	35.55 ± 2.74	35.43 ± 2.36	35.49 ± 3.03
	PC	35.35 ± 2.24	35.73 ± 2.62	35.91 ± 2.21
	TRT1	35.26 ± 2.69	35.68 ± 2.48	35.82 ± 2.41
	TRT2	35.49 ± 2.33	35.78 ± 2.77	35.98 ± 3.46
	TRT3	35.33 ± 2.44	35.71 ± 2.08	35.91 ± 2.44
BUN (mg/dL)	NC	12.23 ± 1.09	12.11 ± 1.15	12.07 ± 1.13
	PC	12.17 ± 1.14	12.50 ± 1.53	12.32 ± 1.17
	TRT1	12.24 ± 0.93	12.38 ± 1.30	12.19 ± 1.44
	TRT2	12.31 ± 1.29	12.40 ± 0.87	55.62 ± 3.68
	TRT3	53.21 ± 4.12	12.34 ± 1.39	55.99 ± 3.16
CREA (mg/dL)	NC	0.79 ± 0.05	0.81 ± 0.05	0.77 ± 0.05
	PC	0.77 ± 0.04	0.85 ± 1.00	0.81 ± 0.07
	TRT1	0.78 ± 0.05	0.83 ± 0.07	0.80 ± 0.06
	TRT2	0.79 ± 0.05	0.80 ± 0.05	0.79 ± 0.08
	TRT3	0.78 ± 0.04	0.79 ± 0.07	0.78 ± 0.06
CPK (U/L)	NC	916.1 ± 40.6	925.4 ± 46.6	945.9 ± 29.1
	PC	917.8 ± 41.9	1170.9 ± 45.9	1245.1 ± 43.6
	TRT1	913.1 ± 33.5	1117.2 ± 44.7 ^a^	1188.5 ± 46.4 ^a^
	TRT2	920.5 ± 42.9	1030.1 ± 43.7 ^c^	1068.7 ± 36.2 ^c^
	TRT3	918.0 ± 42.5	1018.6 ± 40.3 ^c^	991.9 ± 43.1 ^c^

^a,b,c^ Different superscripts within a row indicate a significant difference (*p* < 0.05, *p* < 0.01, *p* < 0.001). ALP, alkaline phosphatase; GLU, glucose; AST, aspartate aminotransferase; ALT, alanine aminotransferase; BUN, blood urea nitrogen; CREA, creatinine; CPK, creatine phosphokinase; NC, negative control; PC, positive control; TRT1, ZA-0.1 (vaccination + 0.1% Amino–Zn dietary intake); TRT2, ZA-0.2 (vaccination + 0.2% Amino–Zn dietary intake); TRT3, ZA-0.3 (vaccination + 0.3% Amino–Zn dietary intake). Sample size n = 20.

## Data Availability

The data presented in this study are available upon request from the corresponding author.
